# The reliability of a restraint sensor system for the computer-supported detection of spinal stabilizing muscle deficiencies

**DOI:** 10.1186/s12891-020-03597-4

**Published:** 2020-09-07

**Authors:** Christian Pfeifle, Melanie Edel, Stefan Schleifenbaum, Andreas Kühnapfel, Christoph-Eckhard Heyde

**Affiliations:** 1 Department of Orthopaedic Surgery, Traumatology and Plastic Surgery, University of Leipzig Medical Center, Liebigstraße 20, 04103 Leipzig, Germany; 2 ZESBO – Centre for Research on Musculoskeletal Systems, University of Leipzig, Semmelweisstraße 14, 04103 Leipzig, Germany; 3 IMISE, Institute for Medical Informatics, Statistics and Epidemiology, University of Leipzig, Härtelstraße 16-18, 04107 Leipzig, Germany

**Keywords:** Autochthonous musculature, BfMC, Computer-supported diagnosis, CTT centaur, Low back pain, Reliability

## Abstract

**Background:**

The presence of muscular deficiency seems to be a major cause of back pain that requires counteractions. Considering that the autochthonous back muscles, responsible for straightening and stabilizing the spine, cannot be activated voluntarily, they can be strengthened only through specific training. The computer-supported test and training system (CTT) Centaur (BfMC GmbH, Leipzig, SN, Germany) seems well suited for this purpose. To show its potential as a reliable diagnostic and training tool, this study aimed to evaluate the test-retest reliability of this 3D spatial rotation device.

**Methods:**

A prospective pilot study was conducted in 20 healthy volunteers of both sexes. For test-retest reliability analysis, three measurements were performed with a two-day interval between each measurement. Each measurement consisted of a one-minute endurance test performed in eight different positions (transverse plane). During the test, the subject was tilted by 90° in the sagittal plane from a neutral, upright position. Meanwhile, the subject’s level of upper body stabilization along the body axis was assessed. All trunk movements (momentum values) were quantified by a multicomponent force sensor and standardized relative to the subject’s upper body mass. The range of motion was assessed by 95% confidence ellipse analysis. Here, all position-specific confidence ellipses for each measurement were merged to a summarized quantity. Finally, ICC analysis using a single-rating, absolute agreement, two-way mixed-effects model and a Bland-Altman plot was performed to determine the reliability.

**Results:**

Considering all measurements (t1, t2, t3), the ICC for reliability evaluation was 0.805, and the corresponding 95% confidence interval (CI) was [0.643, 0.910]. Moreover, the Bland-Altman plots for all three pairs of time points did not show significant differences.

**Conclusion:**

This study concludes that the CTT Centaur shows good test-retest reliability, indicating it can be used in clinical practice in the future.

## Background

Back pain is becoming increasingly common worldwide. It occurs in all age groups and is affected by socioeconomic, environmental, and personal factors [9]. Approximately 85% of back pain complaints are nonspecific, which means that they cannot be directly attributed to injuries or any other identifiable spinal pathologies [4, 5, 8]. However, the presence of muscular deficiency, especially of the autochthonous back muscles, has been considered a possible cause of back pain [7, 10]. Furthermore, pain is commonly associated with a decrease in physical activity [14], which in turn, has adverse effects on the musculature. To counteract this development and reduce low back pain, spinal stabilization exercises, which lead to muscle formation, are highly recommended [16, 19]. As the autochthonous back muscles cannot be activated voluntarily, they are hard to train. However, the Biofeedback Motor Control GmbH (BfMC GmbH, Leipzig, SN, Germany) has addressed this problem and developed the computer-supported spatial rotation trainer “Centaur”. The user is dynamically tilted so that the (autochthonous) back muscles specifically are trained and strengthened [1, 12]. Moreover, muscular deficits are detectable and can be reduced by targeted training. Traditionally, muscle deficit analysis was performed using a tracking system with passive markers. To control the position of the upper body during training, visual markers were previously fixed to the subject and monitored by a camera. Recently, this visual control system has been replaced by a restraint system (shoulder bracket) with an integrated multicomponent force sensor (strain gauges). First, this system should enable more precise position control of the subject when he or she is fixed in the training device. Second, it should guarantee the subject’s safety, as it remains in an upright position in case the subject loses muscular strength during the training. Despite its supposed potential, the reliability of the Centaur as a diagnostic tool has not yet been proven. Instead, previous studies have reported its value in trunk muscle training, which was assessed by surface electromyography (EMG) [1]. This method has been proven to be highly error-prone due to the slippage of electrodes [11], which is why it is not practicable in clinical applications. Thus, EMG analysis of the deep (e.g., autochthonous) muscles is only possible by using needle EMG. This method is also not practicable in clinical practice because it is highly invasive.

The computer-supported test and training device (CTT) Centaur may be appropriate for the analysis of muscular deficiencies and autochthonous muscle training. This study aimed to evaluate its reliability, a measure of reproducibility and one of the main quality criteria for test procedures. Its reliability needs to be verified before the CTT Centaur is considered an alternative diagnostic and training tool in clinical practice.

## Methods

### Participants

Twenty healthy individuals with ages ranging from 21 to 43 years, equally distributed between sexes, were included in this study. As the functionality and safety of the CTT Centaur have not yet been proven, a young and healthy study population was recruited to eliminate confounders such as secondary diseases and minimize unforeseen hazards for the participants. The subjects were recruited from March 2018 to August 2018 using various approaches (e.g., an announcement on our homepage, social networks, public announcements). The volunteers included met all of the inclusion and exclusion criteria (Table 1). These criteria were determined on the basis of the specifications of the certified investigator, local ethics committee, and device manufacturer (BfMC GmbH, Leipzig, SN, Germany). The presence of any contraindications prevented volunteers from participating in the study. To create nearly the same test conditions and reduce the number of confounding factors, the subjects were instructed to maintain a constant lifestyle during the study, especially regarding medication consumption, nutrition and sports participation. Each subject’s health status was verified by a medical examination and anamnesis.

**Table 1:** Inclusion and exclusion criteria

Inclusion Criteria	Exclusion Criteria
Aged 18–75 years	Pregnancy
Body mass ≤ 130 kg	Tumor disease
Participation in all sessions	Recent bone fracture (in the last 6 months)
Constant lifestyle regarding medication consumption,	Recent operation (in the last 6 months)
Nutrition, sports participation, etc.	Findings suggesting the need for acute surgery
	Spinal stiffening operation
	Spinal deformity
	Scar hernia
	Severe vascular disorder
	Severe inflammatory disorder
	Manifest and advanced osteoporosis
	Dementia
	Known heart failure > NYHA Class 2
	Existing therapeutic anticoagulation
	Pre-existing paraesthesia
	Osteoporotic vertebral sintering
	Fresh prolapse of the intervertebral disc
	Pre-existing motor nerve damage
	Dizziness and known kinetosis tendency
	Retinal detachment
	Infectious diseases
	Simultaneous interventional study participation

### Procedure

The study was approved by the local ethics committee (493/16-ek), and each participant gave written informed consent.

**Fig. 1:**
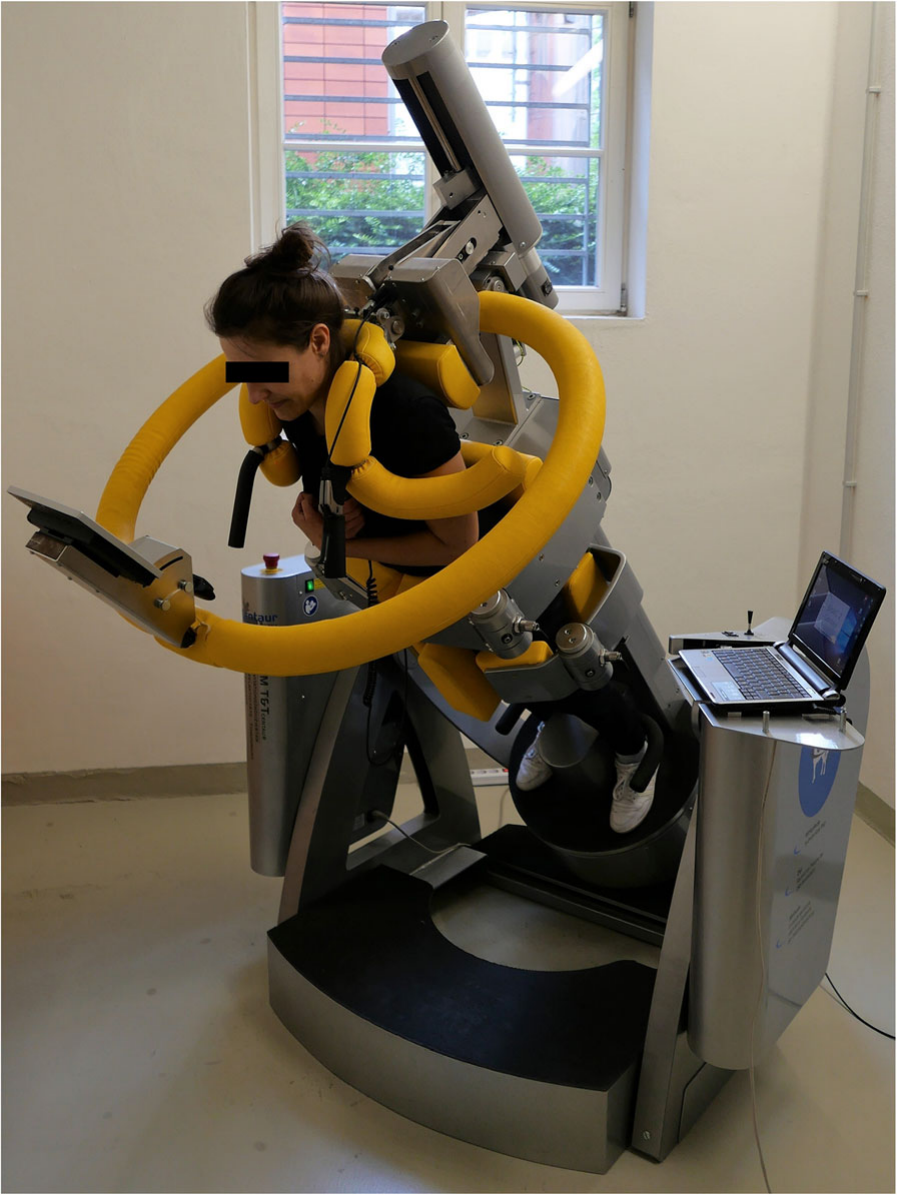
CTT Centaur with the subject tilted in the sagittal plane (transverse plane: 0°)

**Fig. 2:**
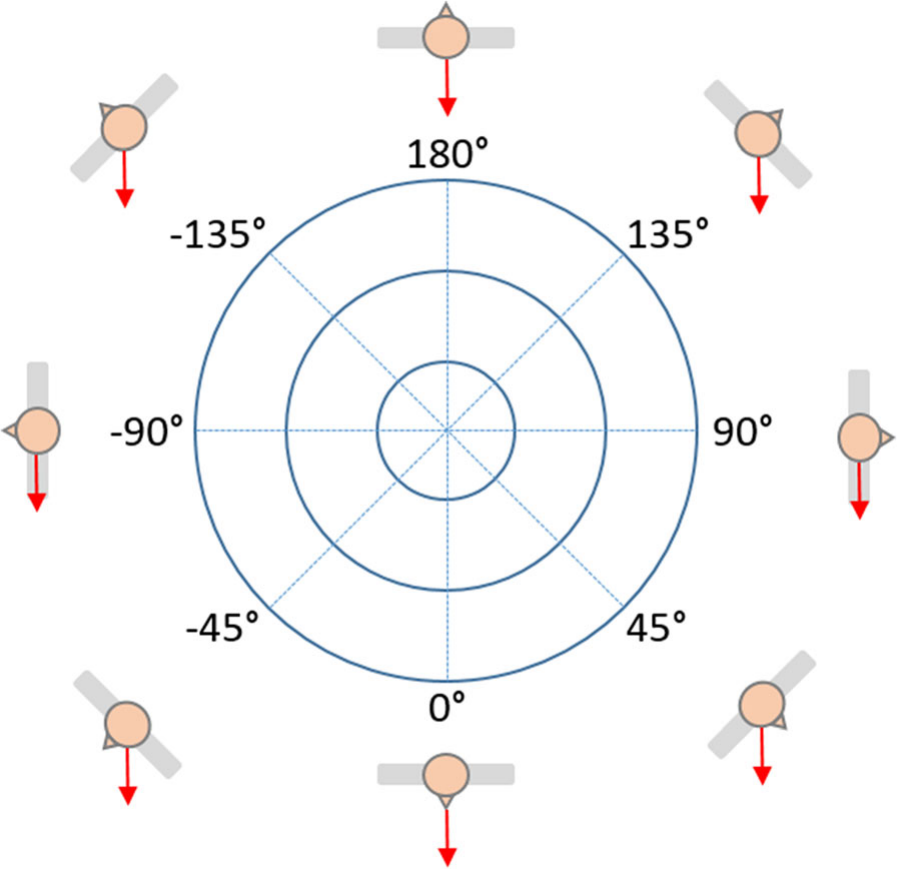
Measurement positions (transverse plane) of the one-minute endurance test (arrow: tilt direction in the sagittal plane)

All tests were conducted in a separate laboratory at the University of Leipzig Medical Center. Within the scope of this prospective study, the test-retest reliability of the 3D spatial rotation device (BfMC GmbH) was evaluated using intraclass correlation (ICC) analysis. Therefore, three measurements (t1, t2, t3) were carried out under the same conditions. The “Centaur” (Fig. 1) is a computer-supported test and training system (CTT) that is used to train autochthonous back muscles by means of applying a precisely reproducible magnitude of strain. After the subject’s lower body is fixed at the hips and thighs, a restraint bracket is positioned over his or her shoulders. When the whole body is tilted in the sagittal plane from the neutral upright position, forces are applied to the unsupported trunk. The subject’s muscle strength was tested by a static endurance test in eight randomized positions (0°, + 45°, − 45°, + 90°, − 90°, + 135°, − 135°, + 180°; “-”: clockwise, “+”: counter clockwise). Figure 2 shows how the subject was oriented in these positions. The red arrows represent the direction of the applied gravitational force. The different positions in the transverse plane were defined by using CTT Centaur software. They were chosen so that strain was applied to all muscle areas in the upper body. The signs (+/−) indicate the Centaur’s direction of rotation. During the test, the participant remained tilted at 90 ° for 1 min at each position. In doing so, the subject permanently tried to stabilize his or her upper body along the body axis against gravitational forces. Meanwhile, a multicomponent force sensor (strain gauges), which was connected with the shoulder restraint system, quantified all movements (x, y coordinates) generated by body contact. These values included momentum values calculated on the basis of the tilt angle, trunk length, patient weight, and force exerted, which was based on the trunk’s deviation from the body axis. By using a feedback system, the movements of the upper body were continuously tracked and displayed dynamically in a coordinate system (x-coordinate: movement in the frontal plane, y-coordinate: movement in the sagittal plane; Fig. 5) to the subject. Thus, the subject could react adequately to adjust his or her posture. Each trial was followed by a total recovery period of 2 min to reduce symptoms of fatigue. During the test, the subject’s arms were crossed in front of the chest. Prior to data collection, the CTT Centaur measurement values were set to zero to calibrate the measurement system. Furthermore, all tests were performed by the same study members, with a two-day interval between the measurements to avoid habituation effects and the impact of aching muscles.

### Data analysis

**Fig. 3:**
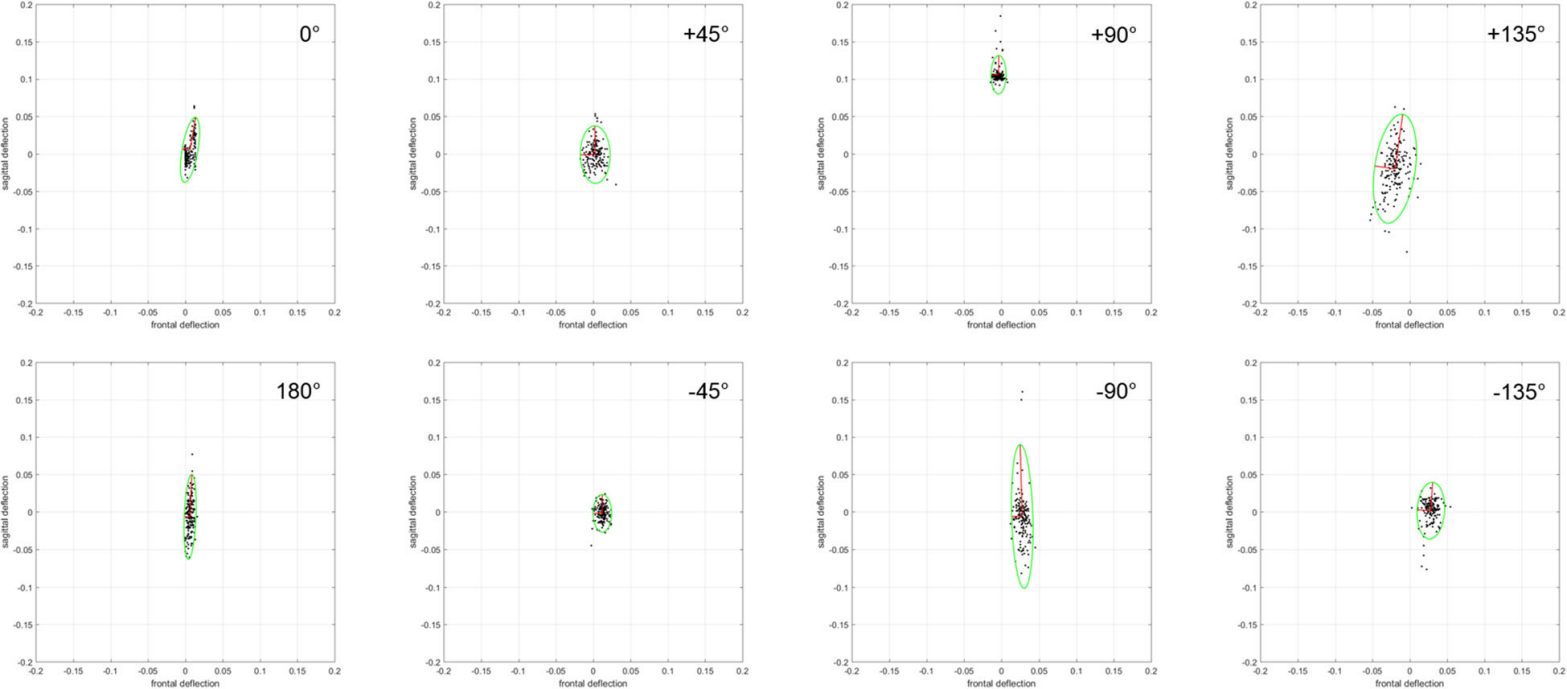
Example of a subject’s upper body movement with the calculated 95% confidence ellipses (green) for one measurement, calculated according to the subject’s position in the transverse plane

After the data collection was completed, the momentum dataset representing the subject’s trunk movement in the frontal plane and sagittal plane was analyzed. Since the momentum values were generated by sensor-trunk contact, all data were standardized (std.) to each subject’s upper body mass (62% of total body mass) [18, 21]. To fully include the sagittal and frontal movements (x, y coordinates), the 95% confidence ellipse (Fig. 3) of each test position was calculated by a custom evaluation procedure using MATLAB, version R2016b (The MathWorks Inc., Natick, MA, USA). These 95% confidence ellipses (Fig. 3) enclosed an area (mm^2^) in which 95% of all movements of the upper body were located. Finally, all confidence ellipses for each measurement were merged to a summarized quantity. This approach allows the whole range of motion to be considered independent of the subject’s spatial orientation, and it is more robust against outliers. Prior to statistical analysis, a data transformation (square root transformation) was performed to obtain normally distributed data and to stabilize the variance. The normality of the data was assessed visually with a histogram and quantitatively with the Shapiro-Wilk test. The ICC estimates and their 95% confidence intervals were calculated using IBM SPSS Statistics, version 23.0 (IBM Corp., Armonk, NY, USA), based on a single-rating, absolute agreement, two-way mixed-effects model (ICC(A,1) [13]; ICC (3, 1) [17, 20]. The statistical significance level was set to be α = 0.05. In this context, values less than 0.50 are indicative of poor reliability, values from 0.50 to 0.69 indicate moderate reliability, values from 0.70 to 0.89 indicate good reliability, and values greater than 0.89 indicate excellent reliability [15]. To determine the level of agreement between different measurements [6], Bland-Altman plots were constructed with the associated limits of agreement (LOA) using SPSS software. Moreover, regression lines including the 95% confidence interval (CI) were added to the Bland-Altman plots. The *p*-values for the effect estimates were also calculated.

## Results

In this study, a group of 20 volunteers, with ten men (m) and ten women (f), was tested. They had a mean age of 29.6 years (± 5.69 years), mean height of 1.77 m (± 0.09 m), mean body mass of 76.0 kg (± 12.32 kg), and mean BMI of 24.2 kg/m^2^ (± 3.43 kg/m^2^) (Table 2).

**Table 2:** Sex-specific characteristics of the study group

	Sex	Minimum	Maximum	Mean	Standard Deviation
Age in years	m	23.0	43.8	31.7	6.31
	f	21.4	37.0	27.5	4.33
Height in m	m	1.80	1.92	1.84	0.04
	f	1.60	1.83	1.70	0.08
Average mass in kg	m	64.0	99.8	81.8	12.05
	f	53.8	83.1	70.2	9.97
BMI in kg/m^2^	m	19.8	29.8	24.2	3.53
	f	19.7	29.9	24.2	3.51

Based on the subjects’ characteristics, the shape and size of the 95% confidence ellipse changed because it was strongly affected by the subjects’ age, upper body mass, athletic condition, and current sensitivities. The characteristics of the resulting merged ellipse areas (mm^2^) are shown in Table 3. Tests on normality yielded nonsignificant results.

**Table 3:** Characteristics of the 95% confidence ellipse areas for each measurement and the results of the normality test (p)

Area ^a^ in mm^2^	Minimum	Maximum	Mean	Standard Deviation	*p*
t1	0.14	0.92	0.5297	0.2221	0.638
t2	0.12	0.82	0.5034	0.2336	0.099
t3	0.14	1.00	0.5336	0.2248	0.732

^a^ Consideration of the sqrt transformed standardized data

The estimated ICC of the square root transformed standardized data for all measurements (t1, t2, t3) was 0.805, and its 95% confidence interval was [0.643, 0.910]. If only two measurements were taken into consideration, the values for the different pairs of time points also indicated good reliability (Table 4). Therefore, the level of reliability for all three measurements can be regarded as good.

**Table 4:** ICC estimates with the 95% confidence intervals for all the pairs of time points

Measurement ^a^	Intraclass Correlation	95%-Confidence Interval	
		Lower Bound	Upper Bound
t1, t2, t3	0.805	0.643	0.910
t1, t2	0.795	0.555	0.913
t1, t3	0.779	0.519	0.907
t2, t3	0.840	0.645	0.933

^a^ Consideration of the sqrt transformed standardized data

**Fig. 4:**
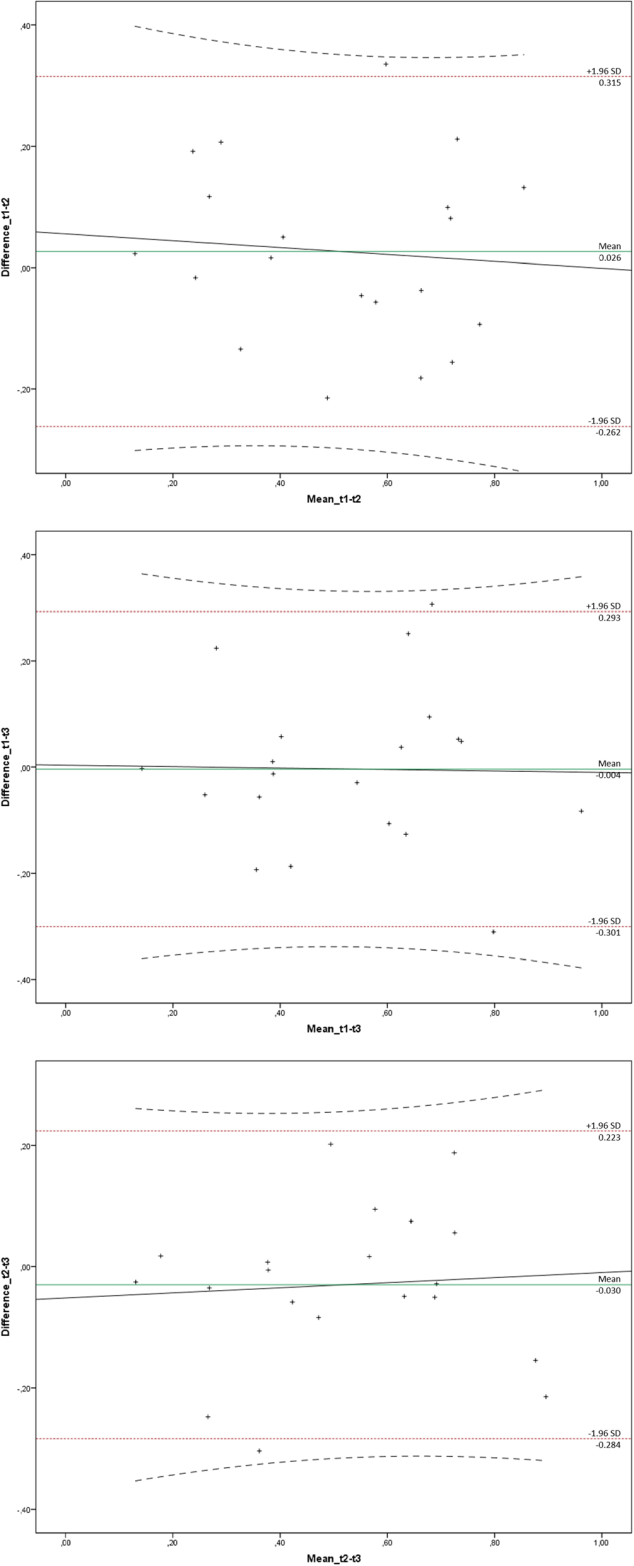
Bland-Altman plots of all the pairs of time points, including the limits of agreement ( ), regression lines (―), and 95% confidence interval (- - -)

The Bland-Altman plots (red: LOA, black: regression line including 95% CI) of all three pairs of time points (t1-t2, t1-t3, t2-t3) are shown in Fig. 4. The results show good reliability, which is also proven by the regression lines and calculated *p*-values (t1,2: 0.728; t1,3: 0.937; t2,3: 0.765) for the effect estimates. Table 5 shows that the mean biases do not differ significantly from each other. Furthermore, the limits of agreement for each measurement varied by only approximately 0.6 mm^2^, which suggests that only marginal differences could be detected [2, 3].

**Table 5:** Bland-Altman analysis

Measurement ^a^	Mean Bias	Standard Deviation	Limits of Agreement (Mean Bias ±1,96 ▪ SD)	
			Lower Limit	Upper Limit
t1, t2	0.026	0.147	−0.262	0.315
t1, t3	−0.004	0.151	−0.301	0.293
t2, t3	−0.030	0.129	−0.284	0.223

^a^ Consideration of the sqrt transformed standardized data

## Discussion

The purpose of this study was to show the reliability of the computer-supported spatial rotation trainer Centaur. According to the ICC calculated using the square root transformed standardized data, there was good concordance. The reliability for each pair of time points for the measurements was qualitatively of the same magnitude, each yielding an ICC of approximately 0.8. The Bland-Altman plots of the pairs of time points also confirmed good reliability, as they did not reveal significant differences (Table 5). Moreover, the plots show no indications of habituation effects within the subjects among the three tests that were carried out.

**Fig. 5:**
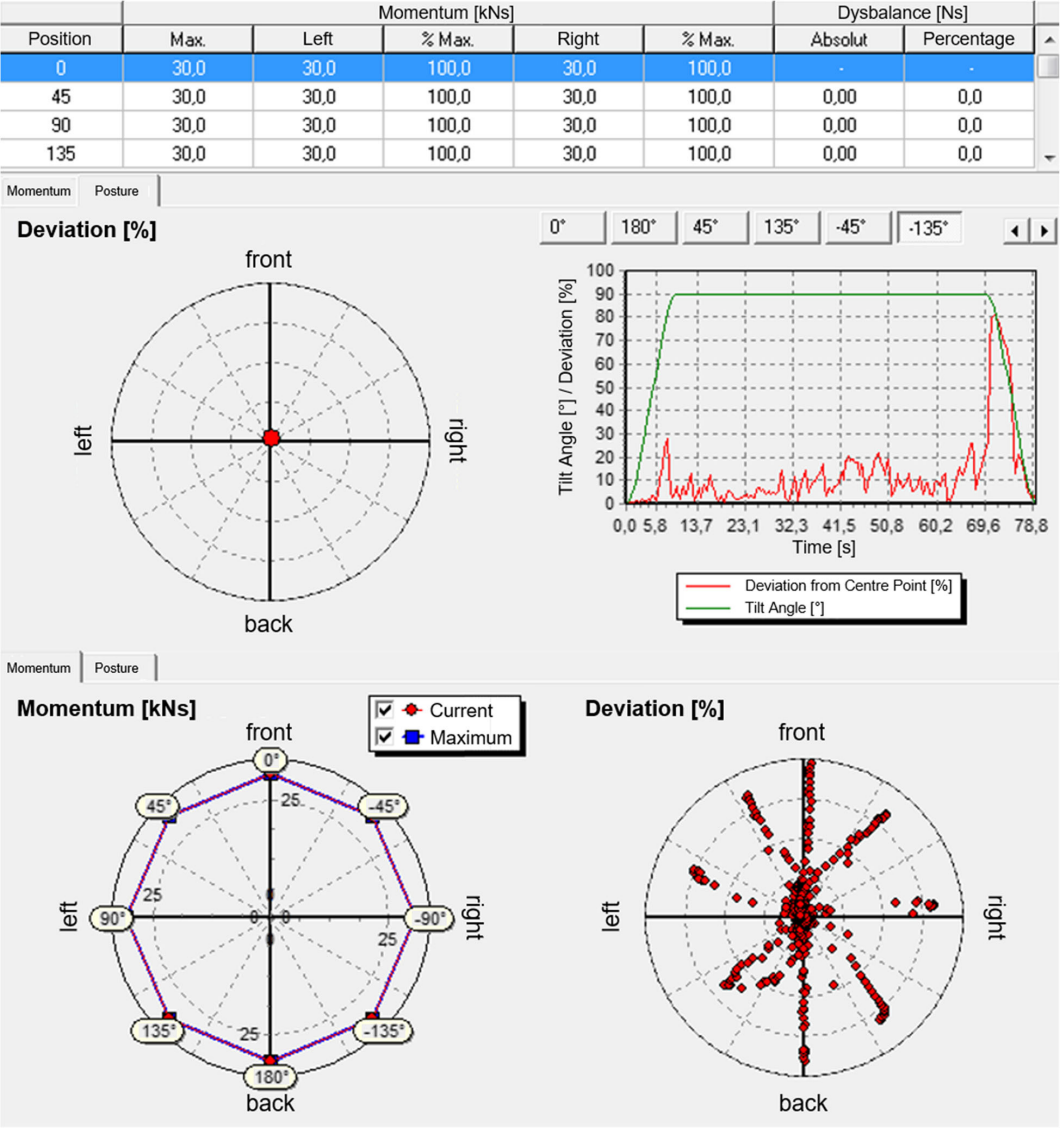
Example of a subject’s movement profile with an interside comparison (upper table), representation of the time-dependent deviation and all upper body movements for one measurement assessed by CTT Centaur software

Since the force sensor registers deviations of the upper body from the body’s longitudinal axis, deficits of functionally connected muscle groups can be registered by increased force exerted and momentum values, respectively. More precisely, an increasing deviation from the zero-point shows increasing deficits of the functional muscle group opposite to the tilt direction. For analytical purposes, these momentum values were recorded by the training device and presented to the therapist in the form of relative values in a graph (Fig. 5). Based on these data, the therapist is able to create specific training programs to strengthen the affected functional muscle group and to reduce back pain.

The standardized data consider the mass of the upper body, which was assumed to be 62% of the total body mass. This value is used in the literature but does not originate from a more precisely specified rounding or average [18, 21].

Moreover, it is never possible to perform the tests under identical conditions at different points in time. Even small differences in factors such as the time of the day and food intake and the onset of a disease can affect the results of the tests. Therefore, a consistently good ICC indicates that the measurement system has high reliability.

Taking these results into account, this measurement system seems to be capable of accurately detecting muscular deficits in the first attempt. For that reason, the CTT Centaur is a reliable diagnostic and training device that can be used to detect and treat spinal stabilizing muscle deficiencies.

## Conclusions

Subjects underwent tests with the CTT Centaur, including the newly developed restraint sensor system, and a reliable analysis of the underlying spinal stabilizing muscle deficiencies was achieved.

A single test does not allow us to draw conclusions about individual muscles. However, the momentum value, tilt angle, and duration of the endurance test can indicate deficits in functionally related muscle groups that contribute to the stabilization of the spinal column. Moreover, interside comparisons can not only provide an overview of the balance of the trunk muscles but also serve as an indicator for medical treatment.

## Limitations

As mentioned before, the tests can never be performed under identical conditions. This is a limitation of the study, but the test conditions represent real conditions in daily clinical practice.

The upper body mass of the tested subjects was estimated to be 62% of their total body mass [18, 21], regardless of their ethnicity, sex, size, physique, etc. People with conditions such as truncal obesity, cachexia, or deviant fat distribution may have a different percentage of upper body mass. However, as shown in the general study group characteristics, the subjects had a BMI between 19.7 kg/m^2^ and 29.9 kg/m^2^, which was not abnormal.

Another limitation of the study is that there was a small number of cases (*n* = 20). Therefore, a larger group of participants is desirable for future studies.

## Data Availability

The datasets used and/or analyzed during the current study are available from the corresponding author upon reasonable request.

## Abbreviations

BfMC: Biofeedback Motor Control
BMWi: Bundesministerium für Wirtschaft und Energie
CI: Confidence interval
CTT: Computer-supported test and training device
EMG: Electromyography
f: Female
ICC: Intraclass correlation coefficient
LOA: Limits of agreement
NYHA: New York Heart Association
m: Male
SD: Standard deviation
sqrt: Square root
t1: First measurement
t2: Second measurement
t3: Third measurement
transf.: Transformed
std.: Standardized
ZIM: Zentrales Innovationsprogramm Mittelstand
